# Safety profile and efficacy of secukinumab in the treatment of autoimmune myasthenia gravis: a single-center retrospective study

**DOI:** 10.3389/fneur.2025.1642938

**Published:** 2025-10-22

**Authors:** Shuangmei Zhang, Xiaodong Song, An Rong Wang, Zhaoxu Zhang

**Affiliations:** ^1^Department of Pain Rehabilitation, Cancer Hospital of the University of Chinese Academy of Sciences (Zhejiang Cancer Hospital), Hangzhou, China; ^2^Institute of Cancer and Basic Medicine (IBMC), Chinese Academy of Sciences, Hangzhou, China; ^3^Department of Neurology, The First Affiliated Hospital of Chongqing Medical University, Chongqing, China; ^4^Department of Traditional Chinese Medicine, The First Affiliated Hospital of Shandong First Medical University & Shandong Provincial Qianfoshan Hospital, Jinan, China; ^5^Department of Neurology, Peking University People's Hospital, Beijing, China

**Keywords:** myasthenia gravis, secukinumab, IL-17, AChR, Th17

## Abstract

**Background:**

Myasthenia gravis (MG) is a chronic autoimmune disease caused by autoantibodies attacking the neuromuscular junction. Traditional treatments are often accompanied by side effects and lack specificity. Recent studies have found that Th17 cells and the inflammatory factor IL-17, which they secrete, play a key role in the pathogenesis of MG and have become potential therapeutic targets. Secukinumab, an IL-17 inhibitor, has shown efficacy in other autoimmune diseases, but its role in MG remains unexplored.

**Objective:**

This study aimed to evaluate the clinical efficacy and immunomodulatory effects of secukinumab in acetylcholine receptor antibody-positive generalized MG (AChR+ gMG).

**Methods:**

In this single-center retrospective study, 29 AChR+ gMG patients received subcutaneous secukinumab (150 mg weekly for 4 weeks, then monthly for 24 weeks). Clinical outcomes (QMG, MG-QOL15, MG-ADL scores), AChR antibody titers, Th17 cell frequency, and IL-17 levels were assessed at baseline and during treatment. Correlations between biomarkers and clinical improvements were analyzed.

**Results:**

By week 24, secukinumab treatment led to significant reductions in disease severity scores (QMG: 60.7%; MG-QOL15: 58.3%; MG-ADL: 64.1%) and AChR antibody levels (69.23%). Th17 cell frequency and IL-17 levels decreased by 68 and 84.47%, respectively. Strong baseline correlations were observed between IL-17, Th17, and clinical scores (*r* = 0.642–0.970, *p* < 0.001), with progressive uncoupling of these relationships during treatment. No severe adverse events were reported.

**Conclusion:**

Secukinumab demonstrated rapid and sustained clinical benefits in AChR+ gMG, linked to suppression of the Th17/IL-17 pathway. These findings highlight IL-17 inhibition as a promising targeted strategy for MG. Limitations include small sample size and retrospective design, warranting validation in larger randomized trials.

## 1 Introduction

Myasthenia gravis (Myasthenia Gravis, MG) is an autoimmune disease affecting the neuromuscular junction, characterized by muscle weakness due to autoantibodies against certain proteins (1, 2). Precision treatment strategies for MG are rapidly evolving, with personalized treatment plans increasingly designed based on the patient’s specific antibody characteristics. Traditional treatments such as immunosuppressants and plasma exchange, while effective to some extent, were not specifically developed for MG and may come with various side effects (3). The successful experience of precision medicine in cancer treatment has provided valuable insights for personalized therapy in MG (4, 5). In recent years, innovative biologics that target key mechanisms such as B cell activation, antibody circulation, and complement system damage at neuromuscular junctions have been proven effective and safe in clinical trials (6, 7).

Th17 cells and IL-17 play a crucial role in the pathogenesis of myasthenia gravis (MG) (8). Studies have shown that an increase in Th17 cells is significantly associated with the severity of MG (9). IL-17, a cytokine secreted by Th17 cells, promotes inflammatory responses and the production of autoantibodies in the pathological process of MG (10). The production of IL-17 is closely linked to the increase in Th17 cells in MG patients, which may lead to the loss of B cell tolerance and the generation of pathogenic antibodies (11). In the experimental autoimmune myasthenia gravis (EAMG) model, inhibiting IL-17 activity can significantly alleviate disease symptoms and reduce the level of anti-acetylcholine receptor (AChR) IgG. Neutralizing IL-17 not only alters the distribution of Th cell subsets but also increases the number of regulatory T cells, indicating that IL-17 plays a crucial role in the immunopathology of MG (12). Our previous studies have also confirmed that the IL-17 level of MG patients is significantly higher than that of the healthy control group and is positively correlated with the baseline severity of MG (13).

The available results suggest that IL-17-based therapeutic strategies may have potential value in the management of MG (14, 15). Secukinumab is currently the most widely used IL-17 inhibitor in global clinical practice, with the richest safety and efficacy data (16, 17). Therefore, we chose it as the investigational drug for this study to preliminarily explore the potential of IL-17 inhibition in the treatment of MG, in order to provide evidence support for targeted therapy of myasthenia gravis.

## 2 Materials and methods

### 2.1 Study population

**Figure 1:**
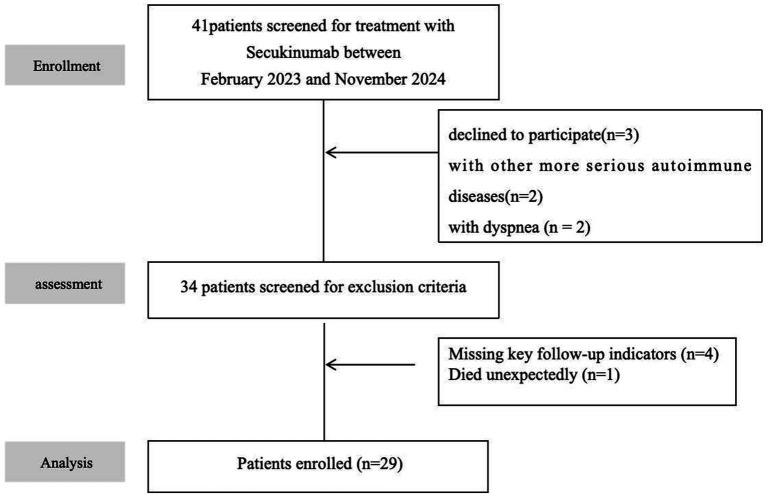
Study flowchart.

This retrospective study enrolled patients diagnosed with myasthenia gravis (MG) at the Department of Neurology, Peking University People’s Hospital, between February 2023 and November 2024. Healthy controls were matched by admission date (Figure 1). Controls had no history of hospitalization or active diseases within the preceding 6 months. After screening, 29 patients were included for statistical analysis and matched with 29 healthy controls (HCs). The two groups were compared in terms of age, gender distribution, disease duration, etc. (Table 1).

**Table 1:** Patient characteristics.

Characteristics	MG (*n* = 29)	HC (*n* = 29)	*p*-value
Age (years), mean (SD)	54.14 ± 14.60	52.07 ± 15.34	0.60
Female, *n* (%)	16 (55.17%)	15 (51.72%)	0.79
Disease duration (years), (SD)	2.48 ± 1.54	–	–
Thymoma (thymectomy), *n* (%)	5 (17.24%)	–	–
Time from Thymoma (years), (SD)	2.28 ± 0.85	–	–
AChR antibody (nmol/L), median (IQR)	25.9(18.3–38.4)	–	–
MGFA type, *n* (%)		–	–
IIa	1(3.45%)	–	–
IIb	11(37.93%)	–	–
IIIа	3(10.34%)	–	–
IIIb	9(31.05%)	–	–
IVa	3(10.34%)	–	–
IVb	2(6.89%)	–	–
MGFA-QMG score, median (IQR)	19(16–24)	–	–
MG-QOL15 score, median (IQR)	20(17–25)	–	–
MG-ADL score, median (IQR)	12(8–15)	–	–

AChR, acetylcholine receptor; MGFA-QMG, Myasthenia Gravis Foundation of America Quantitative Myasthenia Gravis Score; MGQOL-15, 15-item Myasthenia Gravis Quality of Life Scale; MG-ADL, MG-associated Activities of Daily Living score.

### 2.2 Diagnostic and eligibility criteria

The MG diagnosis was confirmed based on clinical history, neurological examination, laboratory assessments, including anti-acetylcholine receptor antibody positivity, and electrophysiological evidence of neuromuscular transmission defects.

Inclusion criteria:

AChR antibody-positive MG;

Aged 18–85 years with MG Foundation of America (MGFA) clinical classification IIa–IVb;

Capacity to provide informed consent.

Exclusion criteria:

Active malignancy (except thymoma);

Severe hepatitis B/C or active tuberculosis;

Severe hepatic/renal insufficiency or multiorgan failure;

Active infections, severe allergies, or pregnancy/lactation.

### 2.3 Intervention

The secukinumab administration regimen adopted in this study (150 MG per week for 4 weeks, followed by 150 MG every 4 weeks for 24 weeks) is a reference to the standard loading dose of secukinumab in autoimmune diseases such as ankylosing spondylitis. The dosages of concomitant therapies (pyridostigmine, corticosteroids, or existing immunosuppressants) were adjusted based on the therapeutic response to secukinumab. A gradual reduction (e.g., of corticosteroids) was allowed upon clinical improvement. No new immunosuppressants were introduced beyond those used at baseline.

### 2.4 Outcome assessments

Peripheral blood samples were collected at baseline, 4, 12, and 24 weeks for antibody titers, cytokine profiling, and flow cytometry analysis (performed strictly per kit protocols) by the Department of Clinical Laboratory (Central Lab) of hospital and any sample with a coefficient of variation (CV) between duplicate measurements exceeding 10% was automatically repeated. Clinical severity was evaluated by two independent neurologists using:

① Quantitative Myasthenia Gravis Score (QMG): 13-item clinician-rated scale (0–39); a 2-point change indicates clinical significance;

② 15-item Myasthenia Gravis Quality of Life Questionnaire (MG-QOL15): Patient-reported outcomes (0–45, higher scores indicate worse quality of life);

③ Myasthenia Gravis Activities of Daily Living (MG-ADL): 8-item patient-reported symptom scale (0–24); a 2-point improvement is clinically meaningful.

In this study, the recognized minimum clinically important difference (MCID) values were adopted: the MG-ADL score and QMG score improved by ≥2 points compared to the baseline, and the MG-QOL15 score improved by ≥6 points compared to the baseline. Baseline data (defined as the last assessment prior to secukinumab initiation) were used to calculate changes in biomarkers and clinical scores.

### 2.5 Safety monitoring

Adverse events (AEs) were monitored via patient-reported symptoms, vital signs, physical examinations, and laboratory tests. AE severity was classified using Common Terminology Criteria for Adverse Events (CTCAE) v5.0, and safety was evaluated descriptively.

### 2.6 Statistical analysis

Continuous variables are expressed as mean ± standard deviation (SD), while categorical variables are summarized as frequency counts and percentages. Nonparametric tests were applied to compare unpaired continuous data (Mann–Whitney *U* test) and categorical variables (Pearson’s chi-square test). The normality of continuous variables was evaluated using the Shapiro–Wilk test. Based on the results, intra-group comparisons were conducted using the parametric paired t-test or the non-parametric Wilcoxon signed rank sum test. *p*-values for all within-group comparisons were corrected with the false discovery rate (FDR) control method (Benjamini-Hochberg procedure). Absolute and relative declines in outcome measures were calculated as follows:

Absolute decline: Baseline value − value at each post-treatment timepoint.

Relative decline: (Absolute decline / Baseline value) × 100%.

Correlations between variables were evaluated using Spearman’s rank correlation coefficient. A two-tailed threshold of *p* < 0.05 defined statistical significance. All analyses were conducted with IBM SPSS Statistics, version 22.0 (IBM Corp., Armonk, NY, United States) and GraphPad Prism, version 9.5 (GraphPad Software Inc., La Jolla, CA, United States).

## 3 Results

### 3.1 Study population and baseline characteristics

This retrospective study analyzed 41 AChR-Ab-positive generalized myasthenia gravis (MG) patients treated with secukinumab at Peking University People’s Hospital between February 2023 and November 2024. After screening out conditions such as concurrent use of other more severe autoimmune diseases, concurrent use of other new biological agents, and lack of key follow-up indicators, 29 patients were included for statistical analysis and matched with 29 healthy controls (HCs). No significant differences were observed in baseline age (MG cohort: 54.14 ± 14.60 years vs. HC: 52.07 ± 15.34 years; *p* = 0.60) or gender distribution (female: 55.17% vs. 51.72%; *p* = 0.79). The MG cohort exhibited a median disease duration of 2.48 years (IQR 1.54), with 17.24% having thymoma history (all resected). Baseline disease severity scores included median MGFA-QMG (19 [IQR 16–24]), MG-QOL15 (20 [IQR 17–25]), and MG-ADL (12 [IQR 8–15]). The baseline demographic and clinical characteristics of the included patients are summarized in Table 1 and Supplementary material 1.

### 3.2 Clinical outcome improvements

**Figure 2:**
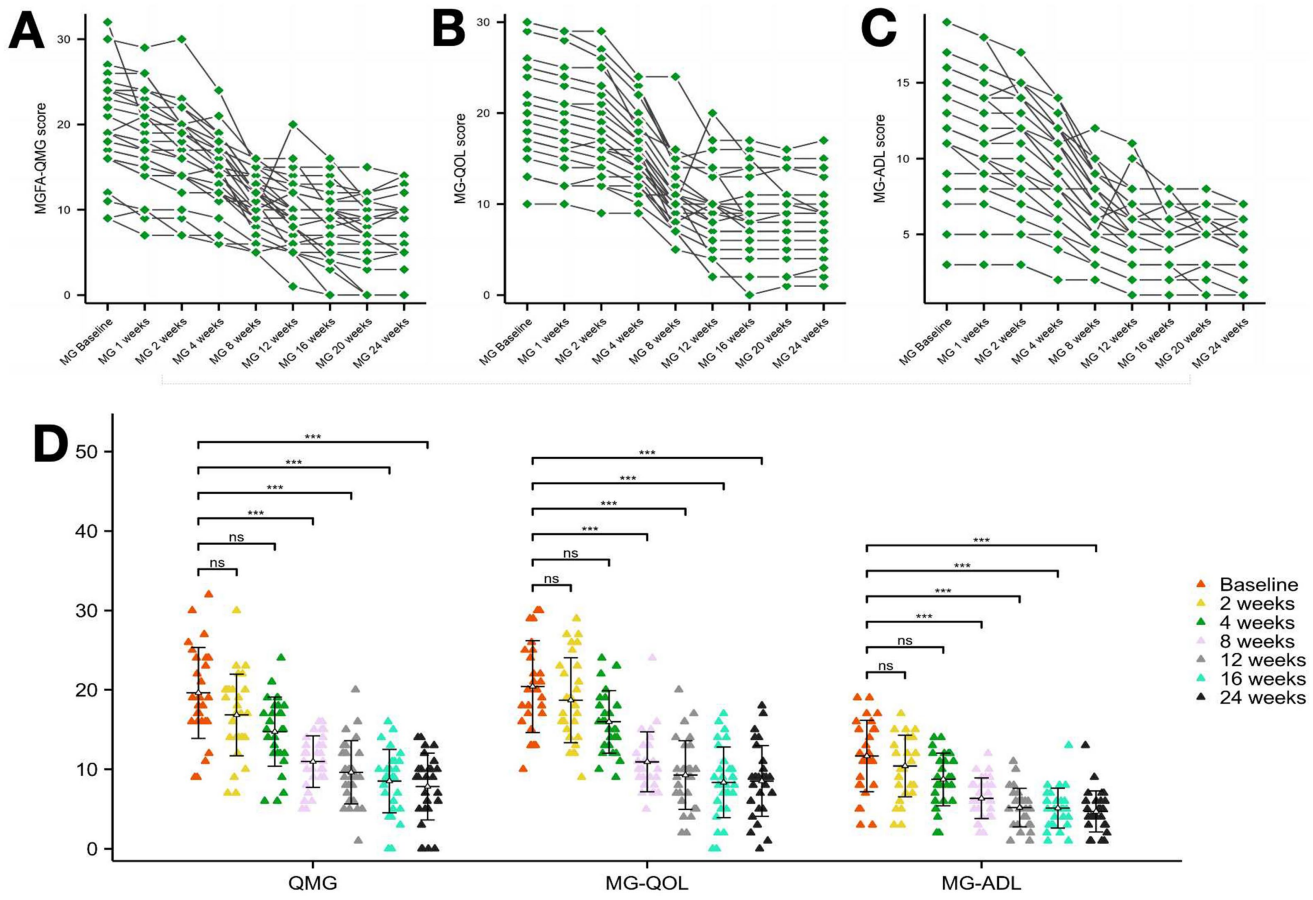
Clinical scale changes following secukinumab treatment from baseline to week 24. **(A)** Change from baseline to week 24 in MGFA-QMG. **(B)** Change from baseline to week 24 in MGQOL. **(C)** Change from baseline to week 24 in MG-ADL. **(D)** Change from baseline to week 24 in MGFA-QMG, MGQOL-15 and MG-ADL. MGFA-QMG, Myasthenia Gravis Foundation of America Quantitative Myasthenia Gravis Score; MGQOL, 15-item Myasthenia Gravis Quality of Life Scale; MG-ADL, MG-associated Activities of Daily Living score. ***p < 0.001.

Secukinumab demonstrated time-dependent therapeutic efficacy across all clinical scales. Initial reductions at 2–4 weeks were non-significant (*p* > 0.05). By week 8, MGFA-QMG scores decreased by 25.0% from baseline (19.6 ± 5.7 vs. 14.7 ± 4.3, *Δ* = 4.9, 95%CI 3.1–6.7, *p* < 0.01), reaching cumulative reductions of 60.7% at week 24. Parallel improvements were observed in quality-of-life (MG-QOL15: 58.3% reduction) and functional capacity (MG-ADL: 64.1% reduction) by study endpoint (Figure 2).

### 3.3 AChR antibody dynamics

**Figure 3:**
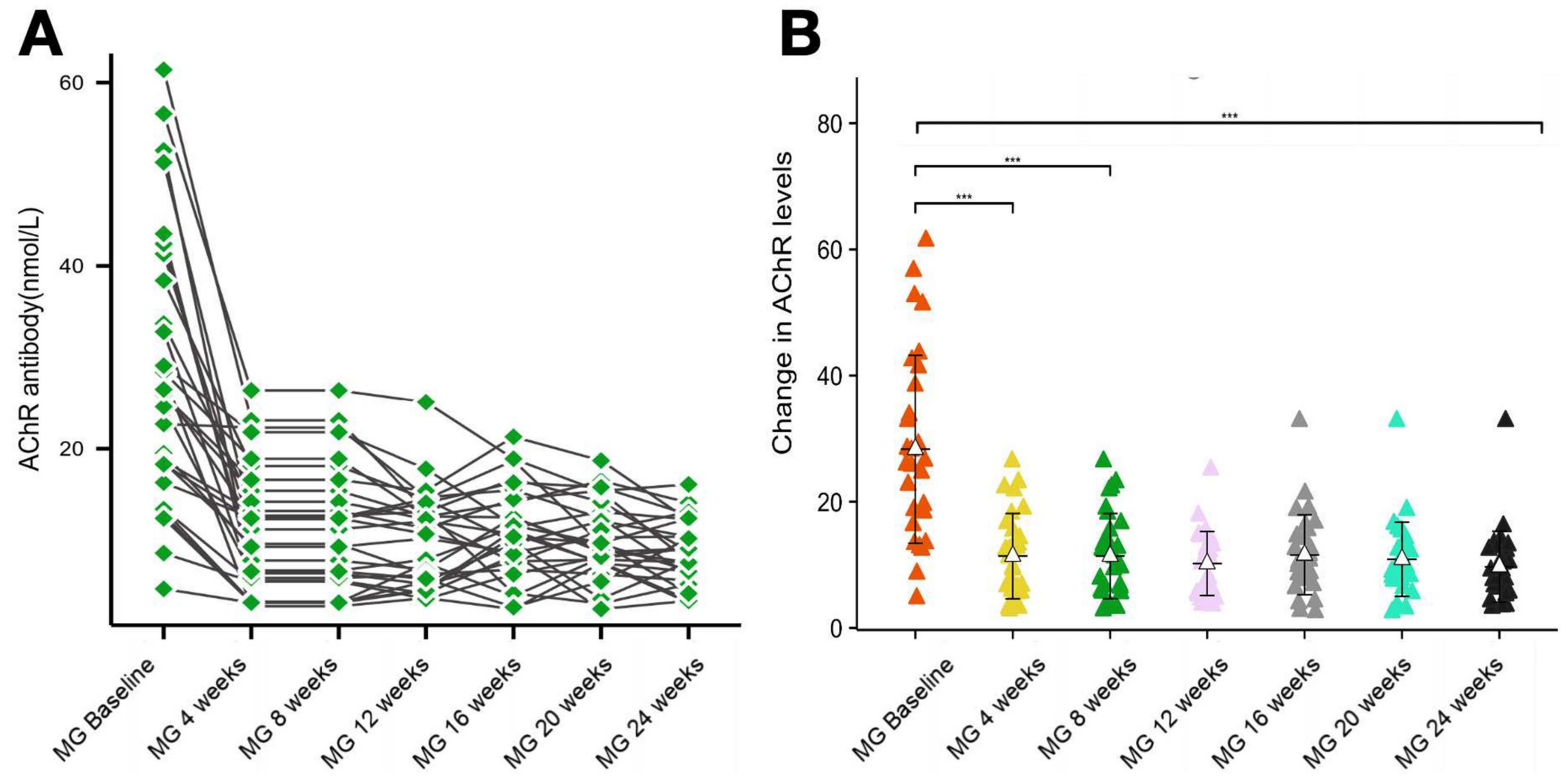
Changes in AChR levels over the study period. **(A,B)** Two graphs are presented to show the changes in AChR levels during the study period. ***p < 0.001.

AChR-Ab levels showed rapid decline post-treatment: 59.80% reduction at 4 weeks and 69.23% cumulative reduction by week 24. Absolute decreases ranged 16.94–19.61 nmol/L from baseline (28.32 nmol/L) to treatment phases (8.71–11.39 nmol/L) (Figure 3).

### 3.4 Th17/IL-17 pathway modulation

**Figure 4:**
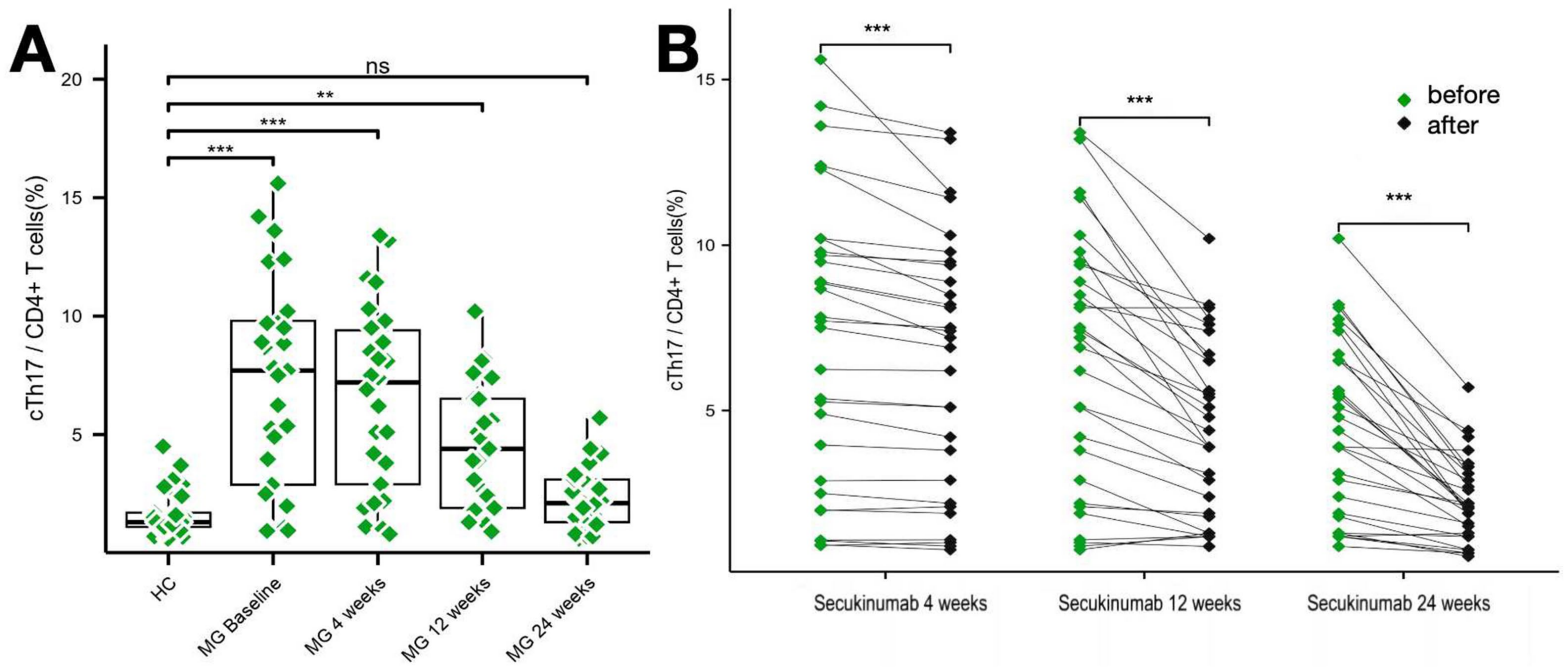
Changes in Th17 cell levels over the study period. **(A,B)** Two graphs are presented to show the changes in Th17 cell levels during the study period. **p < 0.01; ***p <0.001.

**Figure 5:**
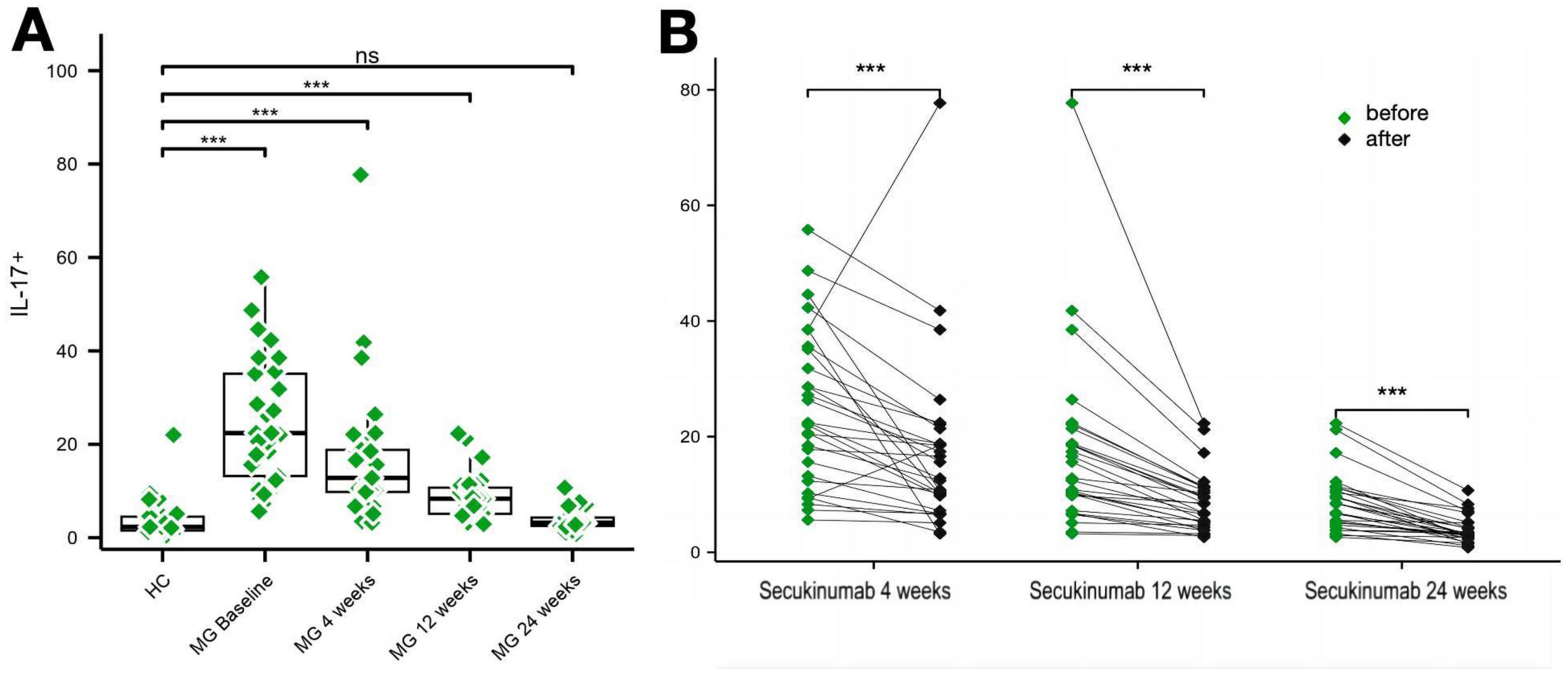
Changes in IL-17 levels over the study period. **(A,B)** Two graphs are presented to show the changes in IL-17 levels during the study period. ***p < 0.001.

Baseline IL-17 (production of CD4 + T cells) levels were significantly elevated in MG patients vs. HCs (24.72 ± 13.46 pg./mL vs. 4.78 ± 7.08 pg./mL, *p* < 0.01), decreasing by 84.47% at week 24 (*p* < 0.001). Th17 (CXCR3 − CCR6^+^ in CD4^+^) cell proportions followed similar kinetics: baseline levels (7.10% ± 4.35% vs. 1.67% ± 0.95% in HCs, *p* < 0.01) reduced to 2.27% ± 1.24% (*Δ* = 68%; *p* < 0.01) (Figures 4, 5).

### 3.5 Individualized heatmap analysis and multi-indicator correlations

**Figure 6:**
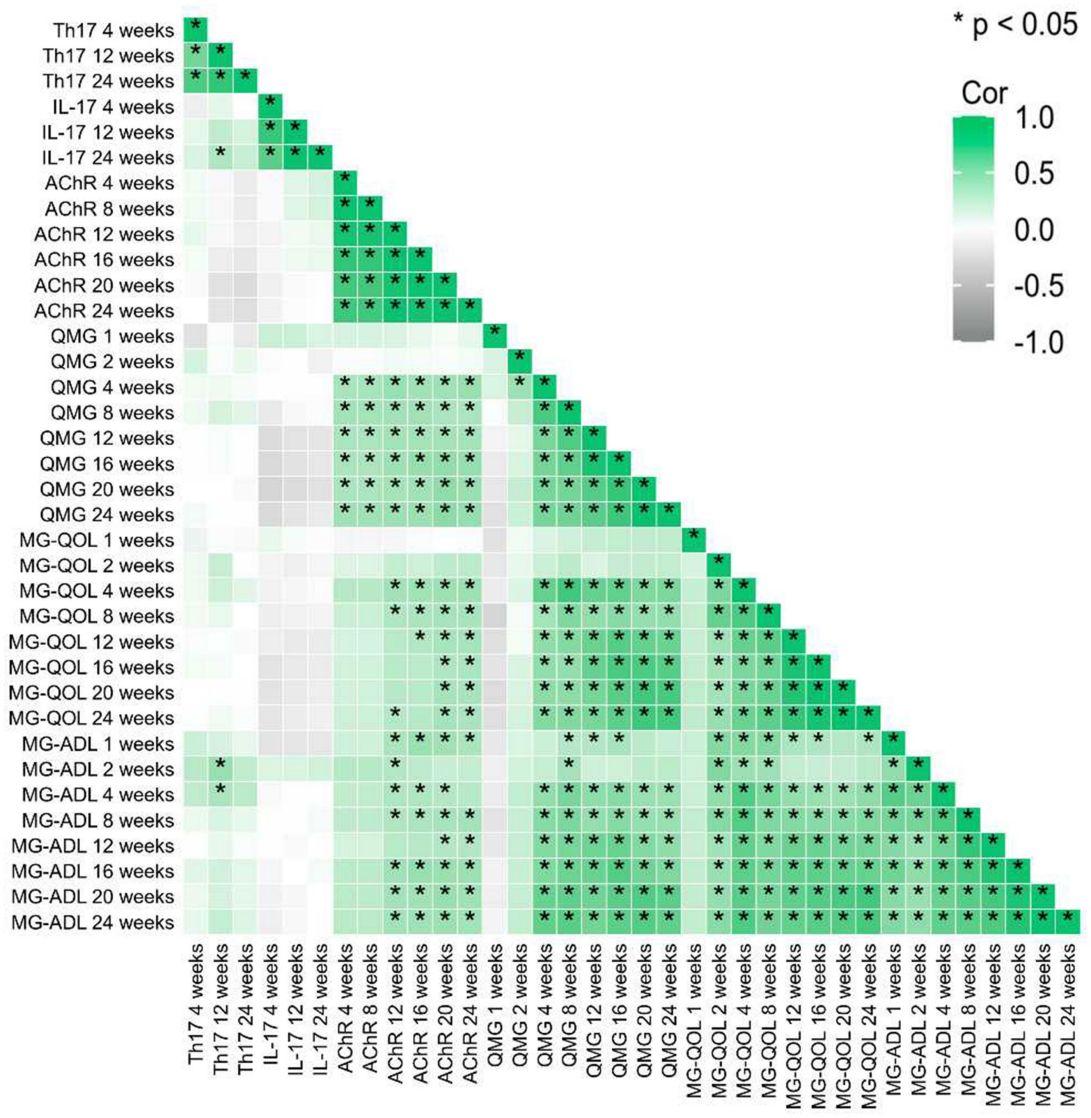
Correlation analysis shows the relationship of relative changes among different time points of each observed factor. * represent *p* < 0.05.

Longitudinal assessments revealed progressive declines in Th17 cell frequency, IL-17 levels, and anti-acetylcholine receptor (AChR) antibody titers over the 24-week treatment course. Concurrently, the Quantitative Myasthenia Gravis (QMG) score, reflecting disease severity, demonstrated a downward trajectory, achieving maximal improvement by week 24 (Figure 6). Patient-reported outcomes, including the Myasthenia Gravis Quality of Life (MG-QOL) and Myasthenia Gravis Activities of Daily Living (MG-ADL) scales, exhibited synchronized optimization, with statistically significant enhancements observed after week 12 (Supplementary material 2).

Baseline correlation heatmaps identified moderate-to-strong positive correlations (*r* = 0.642–0.970, *p* < 0.001) between AChR antibody titers and clinical severity indices (QMG, MG-QOL, and MG-ADL), reinforcing AChR’s role as a biomarker for disease burden.

Dynamic shifts in immune-inflammatory interactions were observed:

Early-phase correlations: At weeks 4 and 12, Th17 cell frequencies showed moderate positive correlations with IL-17 levels (week 4: *r* = 0.536, 95% CI [0.182, 0.761], *p* = 0.003; week 12: *r* = 0.547, 95% CI [0.224, 0.761], *p* = 0.002), indicating coordinated activity during early immunomodulation.

Temporal decoupling: The Th17/IL-17 correlation coefficient attenuated progressively from 0.536 (week 4) to 0.258 (week 24), paralleling reductions in AChR-clinical score correlations (*r* = 0.919 vs. 0.198; *p* < 0.01). The correlation between Th17 cells and IL-17 attenuated progressively from week 4 (*r* = 0.536, 95% CI [0.182, 0.761], *p* = 0.003) to week 24 (*r* = 0.258, 95% CI [−0.126, 0.571], *p* = 0.177). This dissociation implies therapeutic uncoupling of pathogenic Th17-driven inflammation from downstream clinical manifestations (Figure 6).

## 4 Discussion

IL-17 not only further exacerbates the pathological changes in MG by promoting the expression of inflammatory cytokines and inflammatory responses at neuromuscular junctions (15–18), but also disrupts immune tolerance by affecting the function of regulatory T cells (Treg), leading to the immune system’s attack on self-antigens (19). Studies have found that IL-17 can promote the recruitment and activation of inflammatory cells through multiple signaling pathways, thereby exacerbating damage at neuromuscular junctions (20). This retrospective study provides the first clinical evidence supporting the therapeutic potential of secukinumab, an IL-17 inhibitor, in acetylcholine receptor antibody-positive generalized myasthenia gravis (AChR+ gMG). The observed time-dependent improvements in clinical outcomes—60.7% reduction in QMG scores, 58.3% in MG-QOL15, and 64.1% in MG-ADL at week 24—were paralleled by significant declines in pathogenic biomarkers, including AChR antibody titers (69.23% reduction), IL-17 levels (84.47% suppression), and Th17 cell frequency (68% decrease). These findings align with preclinical evidence highlighting the Th17/IL-17 axis as a central driver of MG immunopathology (19, 21, 22), where IL-17 promotes B-cell dysregulation, autoantibody production, and neuromuscular junction inflammation (23). The observed temporal decoupling of Th17/IL-17 correlations from clinical scores (*r* = 0.919 at baseline vs. *r* = 0.198 at week 24) further suggests that secukinumab disrupts the Th17-driven inflammatory cascade, uncoupling immune dysregulation from symptom progression. This mechanistic insight distinguishes secukinumab from complement inhibitors like eculizumab, which target downstream effector pathways rather than upstream T-cell activation. The strong baseline correlations between IL-17 levels, Th17 frequency, and disease severity (QMG: *r* = 0.970; MG-QOL15: *r* = 0.879) align with prior reports (24) implicating Th17 cells in breaking B-cell tolerance and promoting autoantibody production.

Studies have shown that IL-17 not only directly affects B cell function but also indirectly promotes autoantibody production by influencing the balance of T cell subsets (11). These findings position IL-17 as a central therapeutic target, complementing existing strategies focused on B-cell depletion or complement inhibition (25, 26). The sustained improvements in patient-reported outcomes (MG-QOL15, MG-ADL) emphasize secukinumab’s potential to enhance quality of life, a critical endpoint often overlooked in MG trials. This study is the first to directly link IL-17 inhibition to clinical amelioration in human MG, extending prior experimental models (27). The early reduction in AChR antibodies (59.80% by week 4) suggests that secukinumab may accelerate disease modification, offering advantages over conventional immunosuppressants with delayed onset. Furthermore, secukinumab’s subcutaneous administration and favorable safety profile (no severe AEs reported) position it as a viable option for refractory or corticosteroid-dependent patients (27, 28). During the 24-week study period of this study, 2 patients out of 29 had mild skin reactions (redness, pain, or pruritus, which resolved on their own) after injection, and 1 patient had influenza-like symptoms during treatment, no serious adverse reactions occurred in the rest.

The present study provides novel insights into the therapeutic potential of secukinumab, an IL-17 inhibitor, in acetylcholine receptor antibody-positive generalized myasthenia gravis (MG). By demonstrating significant reductions in Th17 cell frequency, IL-17 levels, and clinical severity scores, our findings underscore the pivotal role of the Th17/IL-17 axis in MG immunopathology (21, 29) and highlight secukinumab as a promising targeted therapy. While our study demonstrates a marked reduction in AChR-ab titers following IL-17 inhibition, the precise immunopathological mechanisms—such as the potential effects on total IgG levels, B-cell subset differentiation, and germinal center responses—remain to be fully elucidated. This study has several limitations. First, its retrospective design introduces potential selection bias, and the small sample size (*n* = 29) limits generalizability. Although *post hoc* efficacy analysis indicated that the sample size was sufficient for detecting the observed significant therapeutic effect (Power > 0.8). Second, the absence of a placebo or active comparator group precludes definitive conclusions about secukinumab’s superiority over standard therapies. Third, the 24-week follow-up duration does not address long-term efficacy, safety, or relapse risks. Finally, the exclusion of non-AChR antibody-positive MG subtypes (e.g., MuSK-MG) restricts applicability to a broader MG population. Prospective randomized controlled trials with larger cohorts are needed to validate these findings. Investigations into combination therapies (e.g., secukinumab with complement inhibitors or B-cell-targeting agents) could explore synergistic effects. Longitudinal studies should assess durability of response and long-term safety, particularly regarding infection risks associated with IL-17 inhibition. Additionally, the notable placebo responses observed in high-quality randomized controlled trials for MG, including the ADAPT (efgartigimod) and REGAIN (eculizumab) studies, underscore the need to account for this effect in the design of future clinical investigations (30, 31).

## 5 Conclusion

In summary, our study provides preliminary evidence that secukinumab, by targeting the Th17/IL-17 pathway, may represent a promising novel therapeutic “option” for AChR antibody-positive MG. While limitations inherent to retrospective analyses caution overinterpretation, the robust correlations between biomarker modulation and clinical improvement underscore the translational promise of IL-17 inhibition. These findings warrant further investigation in rigorous clinical trials to establish secukinumab’s role in the evolving landscape of precision MG therapeutics.

## Data Availability

The original contributions presented in the study are included in the article/Supplementary material, further inquiries can be directed to the corresponding author.

## Supplementary material

SUPPLEMENTARY MATERIAL 1 The baseline demographic and clinical characteristics of the included patients.

SUPPLEMENTARY MATERIAL 2 The heatmap demonstrates the relative changes for each individual with MG, relative to baselines.
