# Supplementary material for: Safety profile and efficacy of secukinumab in the treatment of autoimmune myasthenia gravis: a single-center retrospective study
Source: Front Neurol. 2025 Oct 22;16:1642938. doi: 10.3389/fneur.2025.1642938 (PMC12586010; doi:10.3389/fneur.2025.1642938)
Supplement: SUPPLEMENTARY MATERIAL 1 — The baseline demographic and clinical characteristics of the included patients. [file Data_Sheet_1.pdf]

ID	sex	year	Disease duration	Thymoma type	Time from Thymoma (years)	Immunosuppressant	Complications
1	1	48	3	B2	2.3	IVIG, MMF	Post-thymectomy, Chronic gastritis, Colonic polyps, Anxiety/Depression, Thyroid nodule, Arthritis
2	1	68	2.8	B2	2	IVIG, MMF	Hyperlipidemia, Hypertension, Post-thymectomy, Anxiety/Insomnia
3	1	59	1.7	None	0	Tac	Chronic gastritis, Colonic polyps, Prostatic hyperplasia, Anxiety/Insomnia
4	1	52	2.3	None	0	Tac	Hypercholesterolemia, Cervical spondylosis, Anxiety
5	0	37	4.2	None	0	Pred, Tac	Depressive state, Insomnia, Gastric ulcer, Uterine fibroids, Breast nodules
6	0	67	0.8	None	0	MMF, Pred	Diabetes, Benign paroxysmal positional vertigo (BPPV), Migraine
7	0	58	0.5	None	0	MMF, Pred	Breast nodules, Pulmonary nodules, Anxiety
8	1	55	4.2	B2	3	Tac	Post-thymectomy, Insomnia/Anxiety, Hypoalbuminemia, Malnutrition
9	0	74	2.5	None	0	MMF	Hypertension, Diabetes, Carotid plaque
10	0	36	0.4	None	0	MMF, Pred	Sjögren's syndrome, Anxiety/Depression, Migraine
11	1	79	1.6	None	0	MMF	Hypertension, Post-colon cancer resection (stable), Left renal occupying lesion, Hypokalemia, Hyponatremia, Gastroesophageal reflux disease (GERD)
12	0	64	3.2	None	0	HCQ, Pred	Post-thymectomy, Rheumatoid arthritis, Anxiety/Depression, Vestibular vertigo, Cerebral arteriosclerosis
13	0	64	5	A	3.1	IVIG, Pred, Tac	Pulmonary nodules, Hypertension, Insomnia
14	0	29	0.4	None	0	MMF, IVIG	Hyperthyroidism, Anxiety/Depression, Gastric ulcer, Breast nodules
15	1	72	3.4	None	0	AZA, Pred	Hypertension, Prostatic hyperplasia, Arthritis, Anxiety/Depression, Autoimmune hepatitis
16	1	48	2.5	AB	1	AZA, Pred	Post-thymectomy, Gastric ulcer, Anxiety/Depression, Malnutrition, Hypoalbuminemia, Arteriosclerosis
17	0	39	1.6	None	0	IVIG, AZA	Hashimoto's thyroiditis, Insomnia/Anxiety, Cervical spondylosis, Migraine, Peripheral vertigo
18	0	38	2.3	None	0	Pred, Tac	Migraine, Peripheral vertigo, Insomnia/Anxiety
19	1	78	5.6	None	0	AZA, Pred	Hypertension, Hypercholesterolemia, Coronary heart disease
20	0	55	5.2	None	0	Tac, Pred, MMF	Rheumatoid arthritis, Anxiety/Depression, Mild renal insufficiency
21	1	72	2.4	None	0	Pred	Hypertension, Post-esophageal cancer resection (stable), Insomnia/Anxiety
22	1	38	1.2	None	0	Pred	Ankylosing spondylitis, Anxiety/Depression, Gastric ulcer, Ulcerative colitis
23	1	35	3.3	None	0	AZA, Pred	Insomnia, Thyroid nodule, Pulmonary nodule
24	0	63	3.4	None	0	MMF	Hypertension, Cerebrovascular disease, Leukoaraiosis
25	1	59	4.5	None	0	Tac, Pred, MMF	Hypertension, Pulmonary nodule, Gastric polyps, Anxiety/Depression
26	1	37	0.3	None	0	Pred	Insomnia/Anxiety, Thyroid nodule, Pulmonary nodule
27	1	52	1.6	None	0	Pred	Hyperhomocysteinemia, Carotid plaque, Pulmonary nodule
28	1	37	0.4	None	0	Tac, Pred	Peripheral vertigo, Migraine, Insomnia/Anxiety
29	0	57	1.7	None	0	Pred	Hypertension, Insomnia/Anxiety, Ovarian cyst

AZA: Azathioprine； IVIG: Intravenous Immunoglobulin； MMF: Mycophenolate Mofetil； Pred: Prednisone； Tac: Tacrolimus (FK506)
