# Supplementary figures and images for: Safety profile and efficacy of secukinumab in the treatment of autoimmune myasthenia gravis: a single-center retrospective study
Source: Front Neurol. 2025 Oct 22;16:1642938. doi: 10.3389/fneur.2025.1642938 (PMC12586010; doi:10.3389/fneur.2025.1642938)

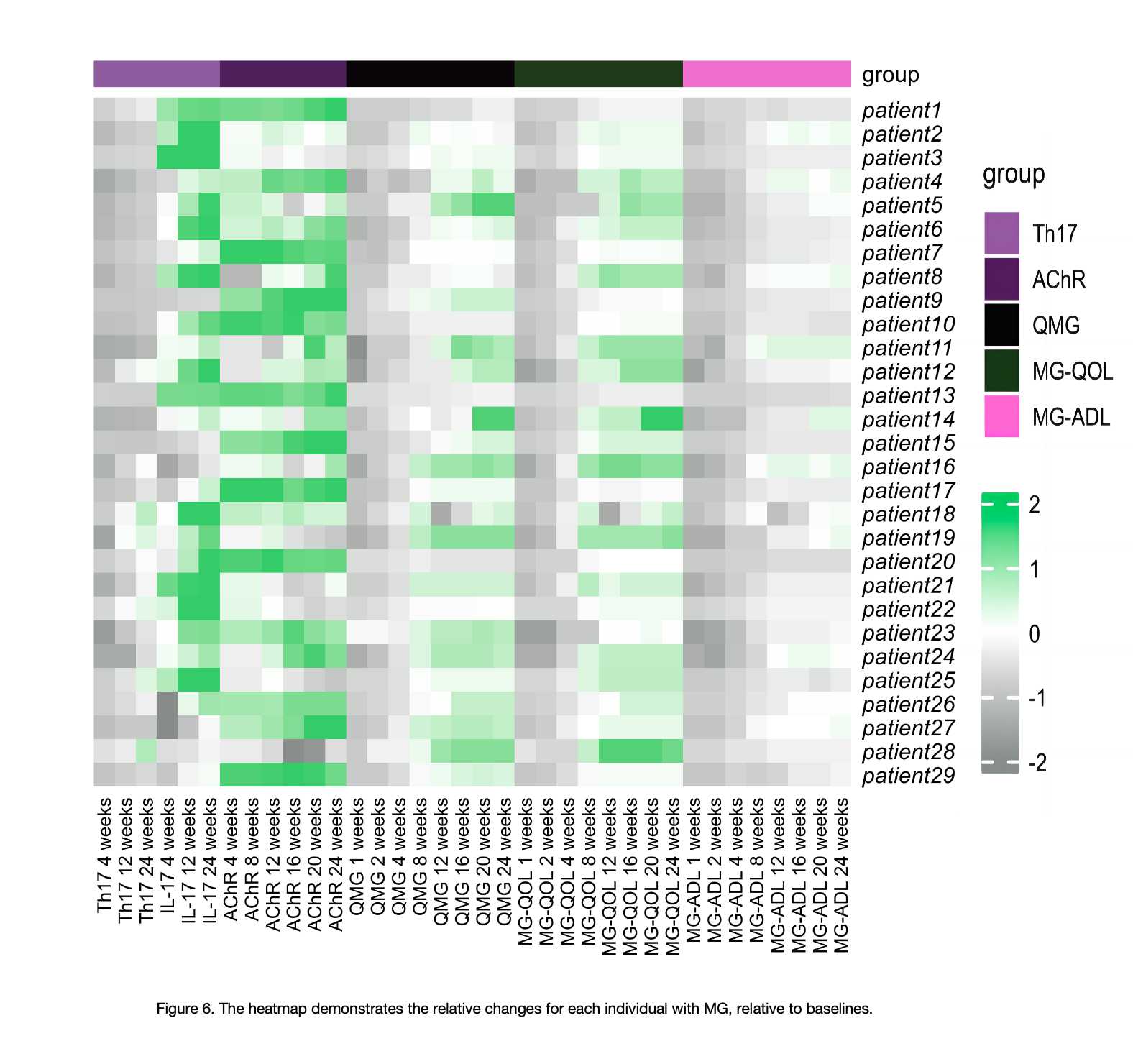

Supplement: SUPPLEMENTARY MATERIAL 2 — The heatmap demonstrates the relative changes for each individual with MG, relative to baselines. [file Image_1.jpeg]
